# HIV Infection Is Associated with Shortened Telomere Length in Ugandans with Suspected Tuberculosis

**DOI:** 10.1371/journal.pone.0163153

**Published:** 2016-09-21

**Authors:** Elizabeth Auld, Jue Lin, Emily Chang, Patrick Byanyima, Irene Ayakaka, Emmanuel Musisi, William Worodria, J. Lucian Davis, Mark Segal, Elizabeth Blackburn, Laurence Huang

**Affiliations:** 1 Division of HIV/AIDS, Department of Medicine, San Francisco General Hospital, University of California San Francisco, San Francisco, California, United States of America; 2 Department of Biophysics and Biochemistry, University of California San Francisco, San Francisco, California, United States of America; 3 Division of Pulmonary and Critical Care Medicine, San Francisco General Hospital, University of California San Francisco, San Francisco, California, United States of America; 4 Makerere University – University of California, San Francisco (MU-UCSF) Research Collaboration, Kampala, Uganda; 5 Department of Medicine, Mulago Hospital, Makerere University, Kampala, Uganda; 6 Department of Pulmonary, Critical Care & Sleep Medicine, Yale University, New Haven, Connecticut, United States of America; 7 Department of Epidemiology and Biostatistics, University of California San Francisco, San Francisco, California, United States of America; University of North Carolina at Chapel Hill, UNITED STATES

## Abstract

**Introduction:**

HIV infection is a risk factor for opportunistic pneumonias such as tuberculosis (TB) and for age-associated health complications. Short telomeres, markers of biological aging, are also associated with an increased risk of age-associated diseases and mortality. Our goals were to use a single cohort of HIV-infected and HIV-uninfected individuals hospitalized with pneumonia to assess whether shortened telomere length was associated with HIV infection, TB diagnosis, and 2-month mortality.

**Methods:**

This was a sub-study of the IHOP Study, a prospective observational study. Participants consisted of 184 adults admitted to Mulago Hospital in Kampala, Uganda who underwent evaluation for suspected TB and were followed for 2 months. Standardized questionnaires were administered to collect demographic and clinical data. PBMCs were isolated and analyzed using quantitative PCR to determine telomere length. The association between HIV infection, demographic and clinical characteristics, and telomere length was assessed, as were the associations between telomere length, TB diagnosis and 2-month mortality. Variables with a *P*≤0.2 in bivariate analysis were included in multivariate models.

**Results:**

No significant demographic or clinical differences were observed between the HIV-infected and HIV-uninfected subjects. Older age (*P*<0.0001), male gender (*P* = 0.04), total pack-years smoked (*P*<0.001), alcohol consumption in the past year (*P* = 0.12), and asthma (*P* = 0.08) were all associated (*P*≤0.2) with shorter telomere length in bivariate analysis. In multivariate analysis adjusting for these five variables, HIV-positive participants had significantly shorter telomeres than HIV-negative participants (β = -0.0621, 95% CI -0.113 to -0.011, *P* = 0.02). Shortened telomeres were not associated with TB or short-term mortality.

**Conclusions:**

The association between HIV infection and shorter telomeres suggests that HIV may play a role in cellular senescence and biological aging and that shorter telomeres may be involved in age-associated health complications seen in this population. The findings indicate a need to further research the impact of HIV on aging.

## Introduction

Widespread use of antiretroviral therapy (ART) has led to a reduction in HIV-related mortality and an increase in the median age of HIV-infected patients worldwide. [1–3] Currently, half of the HIV-infected population residing in the US is ≥50 years of age, [4] with similar trends occurring worldwide, including in sub-Saharan Africa where the vast majority of HIV-infected persons reside. [5] While AIDS-defining illnesses have declined in HIV-infected people with ART-suppressed HIV levels, the incidence of age-associated medical conditions is rising. [1] Interestingly, age-associated conditions often develop in HIV-infected individuals at an earlier age compared to HIV-uninfected individuals. [6] This observation has raised the questions of whether HIV infection is associated with a phenomenon that has been called accelerated biological aging and, if so, what are the precise mechanisms underlying this aging process. As the worldwide HIV population ages, an improved understanding of the association between HIV infection and biological aging is important to the clinical care of HIV-infected persons and to the development of potential therapeutic interventions designed to prevent or slow the progression of these age-associated conditions.

Telomeres are tracts of short DNA sequences complexed with specialized protective proteins, located at the ends of eukaryotic chromosomes, and are subject to shortening during cellular divisions and from DNA damage processes. They preserve genomic integrity by preventing end-fusion and the degradation of chromosomes. [7–9] When telomeres become critically short, they lose their protective functions, and the resulting DNA damage signaling can trigger cellular senescence or apoptosis. [10] The amount and rate of telomere shortening varies among cell types and individuals, with a general trend of older people having shorter telomeres when compared to younger people. [11] In addition to increasing age, a host of factors including stress, [12] low socioeconomic status and education, [13] male gender, [14] alcohol consumption, [15] and environmental exposures such as cigarette smoking [16] have all been shown to negatively affect telomere length. As a measurement of cellular senescence, telomere length is also an indicator of human aging and mortality risk. [17] For example, studies on twins have shown that the twin with shorter telomeres has a three times greater risk of dying first compared to his or her co-twin. [18]

Shortened telomeres have been associated with, and in some cases predict, age-related disorders such as cardiovascular diseases, [4, 19, 20] hypertension, [21] diabetes, [22] and certain types of cancers. [23, 24] Short telomeres are also associated with age-associated pulmonary diseases including pulmonary fibrosis, [25] and chronic obstructive pulmonary disease (COPD). [26] Pneumonia is another age-associated pulmonary disease but the precise association between telomere length and pneumonia is unknown. While chronological age is predictive of both disease development and mortality, telomere length is a marker of biological aging that is thought to reflect predisposition to age-associated diseases independent of chronological age.

HIV infection can lead to chronic immune activation, oxidative stress, and inflammation. [27] HIV is associated with diseases including cardiovascular disease, [28] pulmonary fibrosis, [29] COPD, [30] and cancer, [31] many of which are also associated with age, chronic immune activation, oxidative stress, and inflammation. HIV infection is also correlated with shortened telomeres. [32] Comparing telomere length in HIV-infected and HIV-uninfected individuals may be an important step toward understanding why HIV-infected individuals have a higher incidence of age-related diseases. To date, few data are available that examine HIV infection and telomere length in single cohorts of HIV-infected and HIV-uninfected populations, and fewer studies have examined telomeres in people of African descent living in Africa. [33, 34]

We conducted a prospective study of African patients with and without HIV infection who were hospitalized at Mulago Hospital in Kampala, Uganda with pneumonia and undergoing evaluation for suspected TB. Our goals were to measure telomere length in PBMCs and to examine whether shortened telomere length was associated with HIV infection, TB, and mortality.

## Methods

### Study population and study design

This study was a sub-study of the International HIV-associated Opportunistic Pneumonias (IHOP) Study, a prospective observational cohort focused on HIV-infected patients with pulmonary TB and *Pneumocystis* pneumonia (PCP). [35–40] Participants were excluded from the sub-study if they had hemoglobin levels below 7.0g/dL at the time of enrollment due to ethical concerns related to blood drawing from severely anemic patients. Among eligible subjects, a maximum of two patients per day were selected for the sub-study to allow for the proper collection and processing of blood for telomere measurement. We enrolled 185 adults admitted to Mulago Hospital in Kampala between June 2012 and March 2013.

### Clinical data

We administered a standardized questionnaire and reviewed hospital records to obtain demographic and clinical information. The questionnaire determined current symptoms at time of enrollment, cardiopulmonary comorbidities, and lifestyle questions such as cigarette smoke exposure and alcohol consumption. It also asked about second-hand smoke exposure, both at home and in the workplace, as well as exposure to household cooking smoke. Vital signs were also measured at the time of enrollment.

HIV testing was performed on participants unless prior HIV infection was confirmed. HIV-1 and HIV-2 were screened for using Abbott PCR (Abbott Japan Co ltd. Tokyo, Japan). All positive HIV screening results were confirmed using a STAT-PAK test (Chembio Diagnostic System, INC New York, USA). Patients with a prior positive HIV test were asked questions about their ART use to determine total life-months on ART. CD4 counts were measured on all HIV-infected participants at the time of study enrollment.

Patients were evaluated for TB using a standard approach consisting of two expectorated sputum specimens for LED fluorescence acid-fast bacilli (AFB) smear microscopy (auramine-O staining) and mycobacterial culture (Lowenstein-Jensen media). Those who were sputum smear-negative underwent GeneXpert MTB/RIF testing (Cepheid, Sunnyvale, CA). Those with two negative sputum AFB smears and a negative GeneXpert result were referred for bronchoscopy, which consisted of a visual inspection for Kaposi’s sarcoma lesions and bronchoalveolar lavage (BAL). BAL fluid was sent for AFB smear and mycobacterial culture and microscopic examination for *Pneumocystis*. All subjects were followed throughout hospitalization and were contacted two months after their hospital admission to assess treatment response and vital status. Final diagnosis was assigned two months after patient enrollment using pre-defined, standard criteria, as previously described. [41]

### Specimen collection and laboratory analysis

At time of enrollment, venous blood was collected using an 8 mL BD Vacutainer^®^ Cell Preparation Tube (CPT) with density-gradient polymer gel and sodium citrate additives. Peripheral blood mononuclear cells (PBMCs) were isolated, pipetted into a 15 mL sterile tube with 10 mL of refrigerated Dulbecco’s Phosphate Buffered Saline (DPBS), and then re-centrifuged to wash the cells as previously described. [42] The supernatant was discarded and PBMCs were resuspended in 4 mL of DPBS, divided into 4 Eppendorfs each with 1 ml aliquots, and pelleted using a refrigerated microcentrifuge. PBMC pellets were stored at -80°C and shipped to San Francisco on dry ice for telomere length measurement.

The telomere length measurement assay has been previously described. [43] In short, using a adapted version of Cawthon’s relative measurement of telomere length, telomere length was measured by a quantitative PCR assay that determines the ratio of telomere repeat copy number to single-copy gene copy number (T/S ratio) in participant PBMC samples. [44] To control for inter-assay variability, 8 control DNA samples were included in each run. The average T/S from 10 runs of the same 8 control samples was used as a normalizing factor. The average of the normalizing factors for all 8 controls was used to correct the participant DNA sample results and to get the final T/S ratio. The T/S ratio for each sample was measured twice and averaged. When the two values varied by more than 7%, the sample was run a third time and the two closest values were then used to obtain the average telomere length. The inter-assay coefficient of variation for telomere length measurement in the study is 3.8%. The laboratory personnel who performed the assays received de-identified samples with no knowledge of demographic and clinical data.

### Statistical analysis

Exploratory data analysis revealed one outlier for telomere data (T/S > 2); this individual was removed to leave 184 observations. We compared the medians in continuous demographic and clinical variables between HIV-positive and HIV-negative populations using the rank sum test. Fisher’s exact test was used to compare binary demographic variables.

To evaluate the association of demographic and clinical variables with telomere length, simple regression for continuous variables and t-tests for binary variables were used to estimate effect sizes. We then selected candidate predictors with p-value ≤0.20 to include in our final multivariate model to produce adjusted effects estimates. As our *a priori* stated aim was to examine the association between HIV infection and telomere length, HIV infection was included in multivariate modeling.

Next, we compared the risk of having pulmonary TB and various mortality outcomes with telomere length, adjusted for measures of pneumonia severity (oxygen saturation measured while breathing room air), and overall health (ambulatory status), expressed as odds ratios using logistic regression for binary variables and multiple logistic regression for the multivariate variables. Individuals with an incomplete TB evaluation were excluded from the TB analysis, and those who were lost to follow-up were excluded from the 2-month mortality analyses. All analyses were conducted using R (version 3.0.2).

### Ethics statement

The Mulago Hospital Research Ethics Committee, the Makerere University School of Medicine Research Ethics Committee, the Uganda National Council for Science and Technology, and the Committee on Human Research at the University of California, San Francisco all approved the study protocol. All participants signed written informed consent.

## Results

### Study population

**Fig 1:**
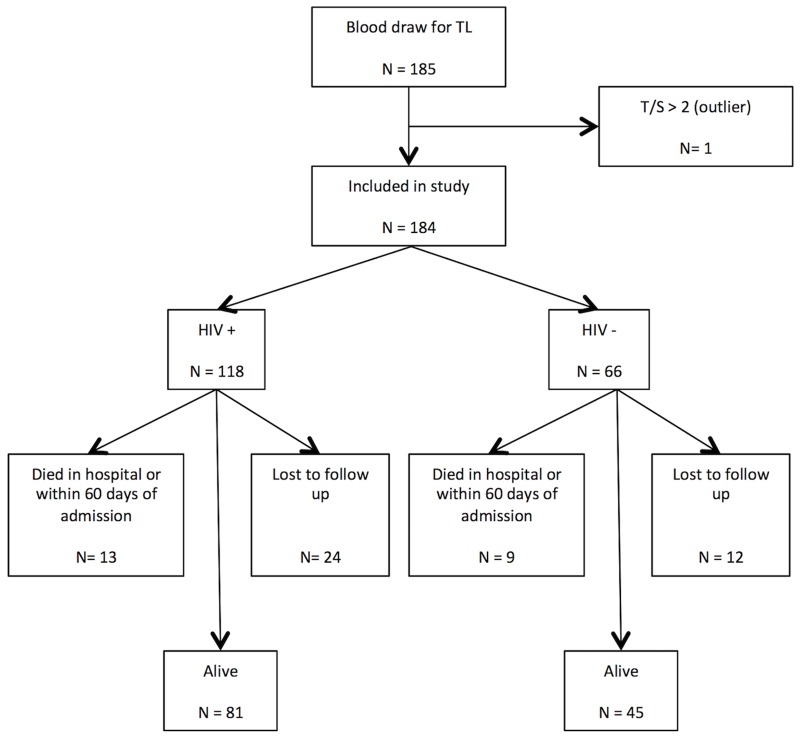
Study population and depiction of mortality after two months of follow up.

Overall, 185 patients were enrolled in the sub-study, and 184 had viable PBMC telomere length results and were included in our analyses (Fig 1). Of these 184 study participants, 118 (64.1%) were HIV-infected and 66 (35.9%) were HIV-uninfected. Characteristics of the study participants are reported in Table 1. Importantly, there were no significant differences in the demographic and clinical characteristics between the HIV-infected and the HIV-uninfected participants, indicating that these individuals were quite similar. All of the participants were of African descent. The HIV-uninfected participants had a median age of 35.6 years, and were on average 2.5 years older than the HIV-infected participants who had a median age of 33.1 years (*P* = 0.58). The majority of the study participants were male, accounting for 60.6% of the HIV-uninfected participants and 52.5% of the HIV-infected participants (*P* = 0.35). Although a minority of the participants reported cigarette smoking and the total amount of cigarettes smoked was low, the HIV-uninfected participants were slightly more likely to have ever smoked cigarettes (27.3% vs. 23.7%, *P* = 0.60) and to be current cigarette smokers (18.2% vs. 16.9%, *P* = 0.84). Similarly, the HIV-uninfected participants were slightly more likely to have had consumed alcohol within the past 12 months (45.5% vs. 38.1%, *P* = 0.35). Comorbidities such as asthma, chronic obstructive pulmonary disease (COPD) and sinusitis were infrequent and no differences were found between the HIV-infected and HIV-uninfected participants.

**Table 1:** Study population characteristics according to HIV status.

Variable	HIV (n = 118)*	no HIV (n = 66)*	p-value
**Overall**			
Age	33.1 (29.3–39.6)	35.6 (25.6–50.5)	0.58
Male sex	52.5 (62)	60.6 (40)	0.35
Ever smoked	23.7 (28)	27.3 (18)	0.60
Currently smoking	16.9 (20)	18.2 (12)	0.84
Pack years	0 (0–0)	0 (0–0.5)	0.56
Secondhand smoke	73.7 (87)	71.2 (47)	0.73
Lived with a smoker?	52.5 (62)	51.5 (34)	1
Alcohol ever	71.2 (84)	65.2 (43)	0.41
Alcohol in last 12 months	38.1 (45)	45.5 (30)	0.35
Telomere length (T/S)	1.02 (0.9–1.1)	1.09 (0.9–1.2)	0.09
Asthma	4.2 (5)	7.6 (5)	0.33
COPD	0.8 (1)	0 (0)	1
Sinusitis	4.2 (5)	3 (2)	1
**Symptoms**			
Fever	84.7 (100)	80.3 (53)	0.54
Weight loss	91.5 (108)	86.4 (57)	0.32
>5 kg weight loss	52.5 (62)	50 (33)	0.76
Sputum	99.2 (117)	100 (66)	1
Dyspnea	50.8 (60)	50 (33)	1
Chest pain	71.2 (84)	65.2 (43)	0.41
Wheezing	26.3 (31)	24.2 (16)	0.86
Bed ridden	22 (26)	25.8 (17)	0.59
**Vital Signs**			
Body temperature	37 (36.2–37.9)	37 (36.3–37.7)	0.80
Heart rate	104.5 (89–122.8)	97 (87–115.8)	0.14
Respiratory rate	26.5 (22–30)	26 (20.3–31.5)	0.27
O2 saturation	97 (95–98)	96 (94–97)	0.01
**Diagnoses**			
2 mo. mortality (n = 148)	13.8 (13)	16.7 (9)	0.64
Discharge mortality	2.5 (3)	3 (2)	1
2 mo. mortality after discharge (n = 148)	10.6 (10)	13 (7)	0.79
Current TB (n = 134)	38.2 (34)	46.7 (21)	0.36
**HIV-only variables**			
CD4 count	144 (35–293)		
Ever on ARV	33.9 (40)		
Currently on ARV	32.2 (38)		
Not currently on ARV	1.7 (2)		
Months on ARV	0.5 (0–7.5)		
Months on current ARV	0.2 (0–7.7)		
New diagnosis	33.9 (40)		

*Results shown as Percent (N) or Median (P25-P75).

Patient symptoms at enrollment including subjective fever, weight loss, cough with sputum, dyspnea, chest pain, wheezing, and being bedridden prior to hospitalization were not significantly different between the HIV-infected and HIV-uninfected patients. Similarly, vital signs at enrollment including temperature, heart rate, and respiratory rate were not statistically different between the HIV-infected and HIV-uninfected patients. Oxygen saturation on room air was statistically different, with the HIV-infected patients having a higher saturation than HIV-uninfected patients (SpO2 97% vs. 96%, *P* = 0.01); however this difference was probably not clinically important. Thus, although none of the demographic variables, symptoms at enrollment, or comorbidities were significantly different in HIV-infected compared to HIV-uninfected participants, the HIV-uninfected group tended to be older, male and cigarette smokers.

Among the 118 HIV-infected participants, HIV infection was newly diagnosed at time of hospitalization in 40 individuals (33.9%) and 38 individuals (32.2%) with known HIV infection were currently receiving ART at time of enrollment (Table 1). CD4 test results were obtained for 117 of 118 HIV-positive participants, with a median CD4 cell count of 144 cells/ul. HIV RNA testing was not performed.

### Diagnoses and mortality

Overall, 50 of the 184 patients had no cause of pneumonia identified, most often due to an incomplete diagnostic evaluation (e.g., patient was discharged prior to completion of entire evaluation). Of the 134 patients with a confirmed diagnosis, TB was the most frequent pneumonia with 55 overall participants being diagnosed with TB. Five (2.7%) participants died while in the hospital, three of these five had HIV infection. Thirty-six participants were lost to follow-up at 2-months. Among the 148 with known vital status at 2-months, an additional 17 participants died, 10 of these 17 were HIV-infected. Compared to the HIV-infected subjects, the proportion of HIV-uninfected participants with a diagnosis of TB (46.7% vs. 38.2%, *P* = 0.36), who died in the hospital (3.0% vs. 2.5%, *P* = 1.00), and who died within two months of hospital admission (16.7% vs. 13.8%, *P* = 0.64) were similar (Table 1).

### Telomere length and predictors of telomere length

The median telomere length, T/S, in the entire cohort was 1.04 (Interquartile Range, IQR = 0.22). Overall, the median T/S in HIV-infected participants was 1.02 (IQR±0.20) and was 1.09 (IQR±0.25) in HIV-uninfected participants (*P* = 0.09). Several factors were significantly associated with shorter telomere length in bivariate analysis (Table 2). Age, gender, cigarette smoking history, alcohol consumption history, and an asthma diagnosis were all associated with telomere length. In contrast, second-hand smoke exposure defined as workplace exposure to cigarette smoke or exposure from living with a cigarette smoker were not significantly associated with shorter telomere length. Similarly, smoke exposure from household cooking was not associated with telomere length. In the bivariate analysis, there was an insignificant trend for HIV infection to be associated with shorter telomere length (*P* = 0.21). Among the HIV-infected participants, new diagnosis of HIV infection and ART use were not associated with telomere length.

**Table 2:** Bivariate and multivariate analysis of predictors of telomere length.

	Bivariate		Multivariate	
	Unadjusted Estimate (95% CI)	p-value	Adjusted Estimate (95% CI)	p-value
Age	-0.0054 (-0.007, -0.003)	<0.0001	-0.0052 (-0.007, -0.003)	<0.0001
Male gender	-0.0539 (-0.106, -0.001)	0.04	-0.0388 (-0.091, 0.014)	0.15
HIV-positive	-0.0346 (-0.089, 0.02)	0.21	-0.0621 (-0.113, -0.011)	0.02
Ever smoker	-0.07 (-0.13, -0.01)	0.02		
Total pack-years	-0.008 (-0.013, -0.003)	<0.001	-0.0037 (-0.008, 0.001)	0.12
Current smoker	-0.0889 (-0.157, -0.021)	0.01		
Current cigarettes/day	-0.0063 (-0.013, 0)	0.047		
2nd hand smoke exposure	-0.0227 (-0.082, 0.036)	0.45		
Ever lived with a smoker	-0.0236 (-0.076, 0.029)	0.38		
Household cooking smoke	0.0131 (-0.161, 0.188)	0.88		
Alcohol ever	-0.0409 (-0.098, 0.016)	0.16		
Alcohol in last 12 months	-0.0422 (-0.096, 0.011)	0.12	-0.026 (-0.077, 0.025)	0.31
Asthma	0.1026 (-0.013, 0.218)	0.08	0.0538 (-0.053, 0.16)	0.32
COPD	0.0113 (-0.348, 0.37)	0.95		
Sinusitis	0.0628 (-0.075, 0.200)	0.37		
**HIV-positive (n = 118)**				
New diagnosis	0.0324 (-0.035, 0.099)	0.34		
Ever on ART (vs. Never)	-0.0012 (-0.069, 0.066)	0.97		

In multivariate analysis, increasing age (β = -0.0052, 95% CI -0.007 to -0.003, *P*<0.0001) and HIV infection (β = − 0.0621, 95% CI -0.113 to -0.011, *P* = 0.02) were both independent predictors of shorter telomere length, controlling for male gender, total pack years of cigarettes smoked, alcohol consumed in the past 12 months, and diagnosis of asthma (Table 2). Since all of the cigarette smoking variables were inter-related, we performed four separate multivariate analyses, each with one of the four smoking variables included along with age, male gender, HIV infection, alcohol, and asthma. In all four analyses, increasing age (*P* = <0.0001 for all) and HIV infection (*P* = 0.016–0.022) remained independent predictors associated with shorter telomere length.

### Telomere length and its association with pneumonia and 2-month mortality

Lastly, we examined the association between telomere length and TB diagnosis and short-term mortality (Table 3), adjusting for severity of pneumonia (oxygen saturation) and overall health status (being bedbound), which were associated with these outcomes in bivariate analyses. No statistically significant correlation was found between telomere length and a diagnosis of TB (*P* = 0.39), or short-term mortality, either in-hospital (*P* = 0.93) or 2-months after admission (*P* = 0.45). Oxygen saturation was associated with both TB (*P* = 0.05) and death within 2-months of admission (adjusted Odds Ratio, aOR = 1.12, *P* = 0.01), but it was not correlated with in-hospital mortality (*P* = 0.78). Patients who were bed bound did not have a statistically significant greater chance of having TB (*P* = 0.11), in hospital mortality (*P* = 0.08), or 2-months post-admission mortality (*P* = 0.23).

**Table 3:** Analyses of association between telomere length and patient status with outcomes.

	TB diagnosis (n = 134)^a^			
	Bivariate		Multivariate	
	Odds Ratio (95% CI)	*p*-value	Adjusted OR (95% CI)	*p*-value
Telomere length*	0.46 (0.068, 2.985)	0.42	0.425 (0.059, 2.960)	0.39
O2 saturation*	1.111 (1.023, 1.232)	0.02	1.095 (1.006, 1.214)	0.053
Not ambulatory	2.45 (1.106, 5.548)	0.03	1.991 (0.863, 4.64)	0.11
	Died in hospital (n = 184)			
	Bivariate		Multivariate	
	Odds Ratio (95% CI)	*p*-value	Adjusted OR (95% CI)	*p*-value
Telomere length*	1.116 (0.009, 187.199)	0.97	1.268 (0.009, 262.893)	0.93
O2 saturation*	1.003 (0.742, 1.163)	0.97		
Not ambulatory	5.213 (0.837, 40.607)	0.08	5.226 (0.838, 40.744)	0.08
	Died within 2 months of admission (n = 148)^b^			
	Bivariate		Multivariate	
	Odds Ratio (95% CI)	*p*-value	Adjusted OR (95% CI)	*p*-value
Telomere length*	0.422 (0.032, 5.528)	0.51	0.35 (0.022, 5.57)	0.45
O2 saturation*	1.122 (1.037, 1.22)	<0.01	1.117 (1.03, 1.218)	0.01
Not ambulatory	2.215 (0.84, 5.661)	0.10	1.824 (0.655, 4.844)	0.23

* Presented in incremental decrease

^a^ Final tuberculosis diagnoses could not be assigned in 50 subjects due to inconclusive test results

^b^ Vital status at 2-months missing in 36 subjects (subjects lost to follow-up).

## Discussion

In this study, we found HIV-infected individuals to have significantly shorter telomeres than HIV-uninfected individuals after controlling for other significant variables. Key strengths of our study are the inclusion of HIV-infected and HIV-uninfected participants who were enrolled from the same cohort with similar demographic and clinical characteristics and a multivariate analysis that controlled for age, gender, cigarette smoking, alcohol consumption, and comorbidities (asthma).

In many past studies, the association between HIV infection and shortened telomeres has been difficult to determine with certainty due to important limitations including the enrollment of HIV-infected and -uninfected participants from similar but separate cohorts or from a failure to control for measured differences in clinical characteristics and exposures between the two populations. In our study, we enrolled all subjects from the same cohort, using the same inclusion and exclusion criteria and thereby minimized the possibility of unmeasured confounders. The absence of significant demographic or clinical differences between the HIV-infected and -uninfected participants supports the similarity of these two groups within our single cohort. The finding that HIV infection was associated with shortened telomere length only after controlling for age, gender, and cigarette smoking, alcohol use, and asthma demonstrates the importance of adjusting for these variables.

Three prior studies have also found a positive association between HIV infection and shortened telomere length, using telomere length as the outcome of interest. [33, 34, 45] Two of the studies use distinctly different cohorts for their HIV-infected and HIV-uninfected participants. One used a single cohort from which they selected both HIV-positive and HIV-negative patients; however, the two groups were not similar in their racial composition or in their alcohol consumption. They also included a significant majority of participants who were women, but the subsequent analysis did not control for gender. Our findings are consistent with these past studies and validate their primary finding of a significant association between HIV infection and shortened telomere length, and importantly we confirmed the results by examining a single homogenous cohort.

There are also two prior studies that fail to show an association between HIV infection and telomere length. [46, 47] The precise explanation for the differing results in these studies and ours is unclear. While our current study validates the significant association between HIV infection and shortened telomeres, the conflicting data in prior studies supports the continued investigation of telomere biology in HIV-infected populations. Our findings are also consistent with past research that has seen increased age, male gender, [14] and cigarette smoking, [16] alcohol, [15] and asthma [48] to be associated with shorter telomeres, adding validity to our finding regarding HIV infection.

Among the HIV-infected group, we did not find a significant association between other HIV-related variables such as CD4 count and ART and telomere length. Prior studies have found conflicting data on the impacts of these HIV-related factors on telomere length. Past studies have found an association between lower CD4 cell counts and higher HIV RNA levels ≥100,000 copies/ml and shorter telomere length. [33, 34] Some studies have shown an association between nucleotide reverse transcriptase inhibitors (NRTIs) and telomere shortening, including two of Uganda’s first line HIV medications, zidovudine (AZT) and tenofovir (TDF). [49–52] Other studies have found no association between antiretroviral treatment and telomere length. [34, 53] Given the life-saving benefits of ART, the aging HIV population, and the increase in age-associated medical conditions in HIV-positive populations, the continued study of the impact of long-term ART and of specific ART regimens on telomere length and telomere biology is critical.

We also did not find a significant correlation between telomere length and TB diagnosis or short-term health outcomes (2-month mortality). While telomere shortening has been associated with increased risk for chronic disease, there is little research on telomere length and acute diseases. Given that telomere shortening occurs over long periods of time, it was not surprising that we did not find a link between telomere length and acute pulmonary infections such as TB or short-term mortality. There was a low prevalence of cardiopulmonary comorbidities among our study population. It is possible that additional study participants had the comorbidities but had not received diagnostic testing to detect their presence. Future studies should examine the association between telomere length and chronic age-associated and HIV-associated comorbidities such as HIV-cardiovascular and HIV-pulmonary diseases using definitive diagnostic tests.

Our study had limitations. First, higher socioeconomic status and education level have been reported predictors of longer telomere length. [54] Our parent IHOP study did not collect these variables, and thus there may have been unaccounted differences between the HIV-infected and HIV-uninfected patients that could affect telomere length. However, past studies conducted at Mulago Hospital have shown no statistically significant difference between HIV-infected and HIV-uninfected patients when comparing occupation and education level, [55] making us confident that these factors were comparable in our HIV-infected and HIV-uninfected groups. We also did not assess duration of HIV illness and duration of ART treatment to understand if those variables affect telomere length. This data could add important insights into our findings and further study is warranted. Additionally, reverse survival bias may have existed given the study population only included sick hospitalized patients. Healthy outpatients were not included in the study. Likely this bias did not affect the results given that both the HIV-infected and HIV-uninfected patients had similar baseline acute illnesses and similar 2-month mortality; however this potential limitation should be taken into consideration. Finally, another limitation is that we did not test for the presence of other chronic viral illnesses such as cytomegalovirus (CMV) in our cohort. CMV infection has been associated with shorter telomere length and reduced telomerase activity and therefore substantial differences in CMV seroprevalence between our HIV-infected and HIV-uninfected subjects might affect our results. [56, 57] One review that included over 25 studies from sub-Saharan African countries found weighted average CMV IgG seroprevalence rates in HIV-infected and HIV-uninfected adult patients to be 80.0% (range 59%-100%) and 79.3% (range 55%-97%), respectively. [58] Thus, the absence of CMV data in our study remains a limitation, but we do not believe that CMV infection is significantly affecting our finding that HIV infection is an independent predictor associated with shorter telomere length due to the similar rates of CMV infection found in numerous studies from sub-Saharan Africa.

HIV infection and short telomeres have both independently been associated with the development of age-associated chronic diseases. Our findings indicate that HIV infection is associated with shortened telomeres, possibly resulting in an increased risk for these chronic conditions which account for a greater proportion of morbidity and mortality in HIV-infected populations. The mechanism by which HIV does this is unknown. Past studies have speculated that HIV causes telomere shortening by decreasing telomerase activity within hematopoietic progenitors. [59] It has also been shown that HIV infection may lead to T-cell replicative senescence, which has been specifically observed in the CD28- CD8+ subset of T-cells. [60] Alternatively, it has been suggested that the oxidative stress associated with chronic inflammation, a process well documented in HIV infection, causes accelerated telomere shortening. [61] Further research assessing these relationships is warranted as the importance of aging in HIV-infected populations is increasing.
